# YL064 activates proteasomal-dependent degradation of c-Myc and synergistically enhances the anti-tumor activity of ABT-199 in diffuse large B cell lymphoma

**DOI:** 10.1038/s41392-020-00236-1

**Published:** 2020-07-06

**Authors:** Huizhuang Shan, Yang Cao, Xinhua Xiao, Meng Liu, Yunzhao Wu, Qi Zhu, Hanzhang Xu, Hu Lei, Zhujun Yao, Yingli Wu

**Affiliations:** 1 Hongqiao International Institute of Medicine, Tongren Hospital/Faculty of Basic Medicine, Chemical Biology Division of Shanghai Universities E-Institutes, Key Laboratory of Cell Differentiation and Apoptosis of the Chinese Ministry of Education, Shanghai Jiao Tong University School of Medicine, Shanghai, 200025 China; 2 Department of Hematology, The Third Affiliated Hospital of Soochow University, Changzhou, Jiangsu Province 213003 PR China; 3 State Key Laboratory for Medical Genomics, Shanghai Institute of Hematology, Collaborative Innovation Center of Hematology, National Research Center for Translational Medicine, Ruijin Hospital, Shanghai Jiao Tong University School of Medicine, Shanghai, 200025 China; 4 Institute of Oncology, Shanghai 9th People’s Hospital, Shanghai Jiao Tong University School of Medicine, 639 Zhizaoju Rd, Shanghai, 200011 China; 5 State Key Laboratory of Coordination Chemistry, Jiangsu Key Laboratory of Advanced Organic Materials, School of Chemistry and Chemical Engineering, Nanjing University, 163 Xianlin Avenue, Nanjing, Jiangsu 210023 China

**Keywords:** Drug development, Target validation

**Dear Editor**,

c-Myc is highly associated with poor prognosis and aggressive progression of diffuse large B cell lymphoma (DLBCL) and is thus a desirable drug target. Moreover, studies indicate that 5–15% of DLBCL patients harbor MYC and BCL-2 translocations, while 20–35% DLBCL patients simultaneously overexpress of c-Myc and BCL-2 proteins without gene rearrangements.^1^ These two types of DLBCL are referred as “double-hit” lymphoma (DHL) and “double-expressor” lymphoma (DEL), respectively. Both DHL and DEL lymphomas have inferior clinical outcomes and are refractory to R-CHOP or even hematopoietic stem cell transplant.^2^ Thus, targeting both c-Myc and BCL-2 is a promising strategy to treat high-risk DLBCLs.^3^ Although BCL-2 inhibitors are clinically available, c-Myc remains to be “undruggable” owing to its lack of kinase activity and intrinsically disordered structure.^4^ Thus, developing clinically applicable c-Myc inhibitor remains challenging.

YL064 is a novel sinomenine derivative (Supplementary Fig. S1a, b) identified in our previous study that inhibits cell growth by targeting STAT3 in multiple myeloma.^5^ However, the effect of YL064 on DLBCL has never been investigated. In this study, we investigated the anti-lymphoma activity of YL064 in DLBCL. As shown in Fig. 1a, YL064 significantly reduced the viability of DLBCL cell lines. When we treated OCI-Ly3 and SU-DHL-2 cells with YL064 or sinomenine (0–10 μM) for 12, 24, and 48 h, YL064 but not sinomenine decreased cell viability in a dose- and time-dependent manner (Supplementary Fig. S2a, b). We further evaluated the influence of YL064 on apoptosis induction and cell cycle progression in DLBCL cells. The treatment of OCI-Ly3 and SU-DHL-2 cells with YL064 significantly increased apoptosis, as evidenced by the increased Annexin V positive cells (Supplementary Fig. S2c) and the substantially increased cleavage of caspase-3, −9 and PARP (Supplementary Fig. S2d). Moreover, YL064 treatment for 24 h increased TUNEL-positive cells in OCI-Ly3 and SU-DHL-2 cells (Supplementary Fig. S2e). Cell cycle analysis showed that YL064-induced G2/M-phase arrest at 12 h (Supplementary Fig. S2f). These results demonstrate that YL064 exhibits anti-DLBCL effect by inducing cell apoptosis and G2/M-phase arrest.

**Fig. 1:**
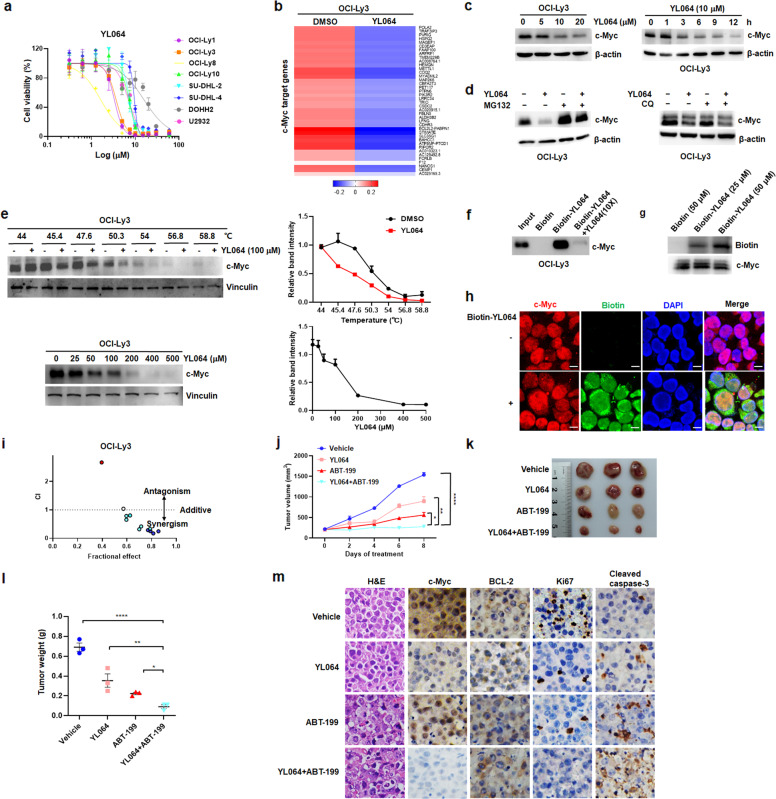
**a** DLBCL cell lines were treated with YL064 at different concentrations for 48 h, and the cell viability was assessed by CCK-8 assay. **b** Heatmap of the top 40 significantly downregulated genes for c-Myc targets in OCI-Ly3 cells treated with YL064 (10 μM) versus DMSO for 6 h. Rows show *Z*-scores are calculated. **c** OCI-Ly3 cells were treated with the indicated concentrations of YL064 for 12 h (left panel) or YL064 (10 μM) for the indicated time points (right panel). Protein expression was determined by western blotting. **d** OCI-Ly3 cells were treated with YL064 (10 μM) in the presence or absence of MG132 (5 μM, left panel) or chloroquine (20 μM, right panel) for 6 h, the indicated proteins were examined by western blotting. **e** The binding between YL064 and c-Myc protein in OCI-Ly3 cells was examined by the CETSA method at different temperatures (upper panel) or doses (lower panel). The protein levels were evaluated by western blotting. The intensity of the c-Myc bands was quantified by Image J software. **f** Biotin-YL064 (50 μM) binding to c-Myc from OCI-Ly3 cell lysate was analyzed after pre-treatment with YL064 (10×). **g** The recombinant c-Myc protein was incubated with biotin-YL064 or biotin for 30 min. The mixtures were subjected to western blotting against biotin or c-Myc. **h** After treatment with or without 10 μM biotin-YL064 for 6 h, OCI-Ly3 cells were stained with c-Myc antibody (c-Myc; red) and streptavidin-FITC (green), followed by counterstaining with DAPI. Scale bars, 10 μm. **i** OCI-Ly3 cells were treated with the indicated concentrations of YL064 and ABT-199, alone and in combination for 48 h. Combination index (CI) for each combination were calculated with the data obtained from the CCK-8 assays with the Calcusyn program. **j**–**m** OCI-Ly3 cells were xenografted in mice as described in supplementary Materials and methods. Mice were treated with vehicle, 30 mg/kg YL064, 50 mg/kg ABT-199, or both drugs. **j** Tumor volume was measured every 2 days. **k** Images of mice (*n* = 3) from each group are presented to show the sizes of the tumors. **l** Tumors were excised from the animals and weighed. **m** Representative H&E staining and immunohistochemical staining of c-Myc, BCL-2, Ki67, and cleaved caspase-3 in tumor sections (original magnification × 400). Data are shown as means ± SEM. *p < 0.05, **p < 0.01, and ****p < 0.0001

In order to reveal the mechanism by which the YL064 induces cytotoxicity in DLBCL cells, two representative DLBCL cell lines OCI-Ly3 and SU-DHL-2 were treated with YL064 for 6 h and subjected to RNA-Seq analysis. Interestingly, heatmaps showed that genes associated with c-Myc targets were dramatically downregulated (Fig. 1b and Supplementary Fig. S3a). Gene set enrichment analysis (GSEA) also uncovered that genes related to c-Myc targets were significantly decreased in YL064-treated cells (Supplementary Fig. S3b). To investigate the role of c-Myc in the anti-DLBCL effect of YL064, we transiently overexpressed c-Myc in OCI-Ly3 and SU-DHL-2 cells (Supplementary Fig. S3c). As illustrated in Supplementary Fig. S3d, e, the overexpression of c-Myc could partially antagonize the anti-proliferative and apoptosis-inducing effects of YL064 treatment. Moreover, the MYC transcriptional expression levels in tested DLBCL cell lines were found to be positively correlated with their susceptibilities to YL064 (Supplementary Fig. S4). These results indicate that c-Myc is involved in the anti-DLBCL effect of YL064.

Based on the fact that c-Myc plays an important role in YL064-mediated growth inhibition of DLBCL cells, we next sought to determine the effect of YL064 on the expression of c-Myc. YL064 markedly reduced the protein levels of c-Myc in OCI-Ly3 and SU-DHL-2 cells in a dose- and time-dependent manner (Fig. 1c and Supplementary Fig. S5a), while sinomenine did not induce a similar effect (Supplementary Fig. S5b). Moreover, MYC and MAX mRNA levels were not obviously changed following YL064 treatment (Supplementary Fig. S5d), indicating that the reduction of c-Myc protein occurs at the post-transcriptional level. Interestingly, the presence of a proteasome inhibitor MG132, rather than lysosome inhibitor chloroquine (CQ), prevented the YL064-induced c-Myc degradation (Fig. 1d and Supplementary Fig. S5c). Moreover, YL064 treatment shortened the half-life of c-Myc protein (Supplementary Fig. S5e). To further confirm that YL064 induces c-Myc degradation through the ubiquitin-proteasome system, we transfected HEK293T cells with plasmids encoding Flag-c-Myc and found that the ubiquitination level of c-Myc increased after YL064 treatment (Supplementary Fig. S5f). In addition, YL064 treatment selectively increased c-Myc threonine 58 (T58) phosphorylation, but not that of serine 62 (S62) (Supplementary Fig. S5g), indicating the involvement of c-Myc phosphorylation in YL064-mediated c-Myc degradation. Furthermore, the addition of MLN4924, an indirect inhibitor of cullin-RING E3 ligases by blocking cullin neddylation, blocked the degradation of c-Myc mediated by YL064 in OCI-Ly3 and SU-DHL-2 cells (Supplementary Fig. S6). These results suggest that YL064 induces ubiquitin-proteasome-dependent degradation of c-Myc in DLBCL cells.

To uncover the molecular basis for the inhibition of c-Myc by YL064, we sought to verify whether c-Myc protein is a cellular target of YL064. For this purpose, cellular thermal shift assay (CETSA), a newly developed approach based on the thermal stabilization of ligand-bound proteins, was performed on OCI-Ly3 and SU-DHL-2 cells. Compared with the DMSO-treated group, YL064 treatment markedly decreased the thermal stability of c-Myc protein at different temperatures (Fig. 1e, upper panel and Supplementary Fig. S7a). Furthermore, the thermal stability of c-Myc protein was tapered by YL064 in a dose-dependent manner (Fig. 1e, lower panel and Supplementary Fig. S7b). These data indicate that YL064 may interact directly with c-Myc in cells. To further confirm this hypothesis, biotin-labeled YL064 was used to conduct the pull-down assay. As depicted in Fig. 1f, biotin-YL064 effectively pulled down c-Myc from the cell lysate. Immunofluorescence staining also showed that biotin-YL064 was co-localized with c-Myc in the nucleus of OCI-Ly3 cells (Fig. 1h). To further validate the direct interaction of YL064 and c-Myc, we incubated biotin-YL064 with recombinant c-Myc protein in vitro, and found that biotin-YL064 directly bound to c-Myc in a dose-dependent manner (Fig. 1g). More importantly, this interaction was competitively inhibited by ten-fold concentrations of unlabeled YL064 but not by sinomenine (Supplementary Fig. S7c). We subsequently mapped the domains of c-Myc that might be responsible for its interaction with YL064 (Supplementary Fig. S7d, upper panel). Using the immunoprecipitation assay, we found that the C-terminal HLH-Zip domain of c-Myc (amino acids 368–439) was crucial for interaction with YL064 (Supplementary Fig. S7d, lower panel). These data provide compelling evidence supporting that YL064 interacts directly with c-Myc in DLBCL cells.

Since the high expressions of both c-Myc and BCL-2 contribute to a worse outcome in DHL and DEL than in other DLBCL subtypes, we hypothesized that targeting c-Myc and BCL-2 simultaneously by YL064 and ABT-199, a clinically available BCL-2 inhibitor, may be more effective in inducing cell death of DLBCL cells. A distinctly synergistic effect was observed in both DHL (OCI-Ly1 and OCI-Ly8) and DEL (OCI-Ly3 and U2932) lymphoma cell lines (Fig. 1i and Supplementary Fig. S8a, b, d). CI values of YL064 and ABT-199 in DLBCL cells were shown in Supplementary Table S1. Of note, a slightly synergistic or even antagonistic effect of YL064 and ABT-199 in SU-DHL-2 cells was observed (Supplementary Fig. S8c). Therefore, we sought to examine whether YL064 affects the protein levels related to the BCL-2 family. We found that YL064 treatment reduced the protein levels of MCL-1 but had no effect on other proteins such as BIM, BAX, and BCL-xL (Supplementary Fig. S9). Interestingly, YL064 treatment decreased BCL-2 protein levels in SU-DHL-2 but not in OCI-Ly3 cells (Supplementary Fig. S9). The removal of ABT-199 target may explain why the synergistic effect is not obvious in SU-DHL-2 cells. Next, we also examined whether the co-treatment of YL064 and ABT-199 could enhance YL064-induced growth inhibition or apoptosis in OCI-Ly3 cells. As expected, ABT-199 enhanced the cytotoxicity of YL064 (Supplementary Fig. S8e). Moreover, in OCI-Ly3 cells, the ratio of Annexin V positive cells was significantly increased after treated with YL064 plus ABT-199 compared with the groups treated with a single drug (Supplementary Fig. S8f). Taken together, these observations set the stage for the potential therapeutic application of YL064 in combination with ABT-199.

Consistent with our previous studies,^5^ YL064 also inhibited the phosphorylation of STAT3 on Tyr705 in DLBCL cells without affecting the total level of STAT3 (Supplementary Fig. S10a). Heatmaps showed that genes associated with the STAT3 targets were dramatically downregulated (Supplementary Fig. S10b). In order to evaluate the effect of STAT3 on the combined effect of YL064 and ABT-199, we used a selective STAT3 inhibitor, Stattic, to treat DLBCL cells. Stattic markedly inhibited the phosphorylation of STAT3 on Tyr705 and suppressed the proliferation of OCI-Ly3 cells (Supplementary Fig. S10d, e). Although Stattic could enhance the effect of ABT-199 to some degree, no synergistic anti-proliferative effect was obtained in OCI-Ly3 cells (Supplementary Fig. S10f). These results indicate that the inhibition of STAT3 may not play a key role in the combination effect of YL064 and ABT-199. We further compared the target genes of c-Myc and STAT3 in the downregulated genes of RNA-Seq data (Supplementary Fig. S10c). Since the combination of STAT3 inhibitor with ABT-199 has no synergistic effect on DLBCL cells, we proposed that the c-Myc specific target genes may play an essential role in the combination effect of YL064 plus ABT-199 (Supplementary Table S2).

In order to test the synergistic effect of YL064/ABT-199 combination towards high-risk DLBCLs in vivo, we chose OCI-Ly3 as a model of DEL lymphoma and OCI-Ly8 as a model of DHL lymphoma, respectively. NCG mice bearing subcutaneous xenografts of OCI-Ly3 cells were randomly divided into four treatment groups to receive YL064 (30 mg/kg), ABT-199 (50 mg/kg), their combination, or the corresponding vehicle. The results showed a reduction in tumor volumes in mice with combined treatment, as compared with YL064 or ABT-199 treatment alone (Fig. 1j). At the end of the treatment, the mice from each group were sacrificed, whose tumors were removed, photographed, and weighed. As displayed in Fig. 1k, l, the tumor size and tumor weight of the combination-treated group were lower than those of the other groups. Furthermore, the immunohistochemical (IHC) analysis revealed a markedly decrease in the cell proliferation marker Ki67, while the cell apoptosis marker cleaved caspase-3 was increased in the combination-treated tumors (Fig. 1m). More importantly, we also observed prominent downregulation of c-Myc after co-treatment, whereas BCL-2 was not changed (Fig. 1m). Similar results were obtained in the OCI-Ly8 xenograft model (Supplementary Fig. S11). These results suggest that YL064, in combination with ABT-199, is more effective compared with monotherapy in treating DLBCL in vivo.

In summary, our data support that YL064 is a promising compound for the treatment of DLBCL through directly targeting c-Myc for degradation. Moreover, we demonstrated the combination of a c-Myc degrader and a BCL-2 inhibitor exerts synergistic effectiveness against DLBCL. Further elucidating the interaction relationship between YL064 and c-Myc may pave the way for the development of novel c-Myc-specific degraders for the treatment of DLBCL.

## Supplementary information

Supplementary Information
