# Supplementary material for: YL064 activates proteasomal-dependent degradation of c-Myc and synergistically enhances the anti-tumor activity of ABT-199 in diffuse large B cell lymphoma
Source: Signal Transduct Target Ther. 2020 Jul 6;5:116. doi: 10.1038/s41392-020-00236-1 (PMC7338474; doi:10.1038/s41392-020-00236-1)
Supplement: Supplementary file 1 — Supplementary Information [file 41392_2020_236_MOESM1_ESM.docx]

Supplementary Materials for

YL064 activates proteasomal-dependent degradation of c-Myc and synergistically enhances the anti-tumor activity of ABT-199 in diffuse large B cell lymphoma

Huizhuang Shan^1^, Yang Cao^2^, Xinhua Xiao^3^, Meng Liu ^1^, Yunzhao Wu^1^, Qi Zhu^4^, Hanzhang Xu^1^, Hu Lei^1^, Zhujun Yao^5*^, Yingli Wu^1*^

^1^ Hongqiao International Institute of Medicine, Tongren Hospital / Faculty of Basic Medicine, Chemical Biology Division of Shanghai Universities E-Institutes, Key Laboratory of Cell Differentiation and Apoptosis of the Chinese Ministry of Education,

Shanghai Jiao Tong University School of Medicine, Shanghai 200025, China; ^2^ Department of Hematology, The Third Affiliated Hospital of Soochow University, Changzhou, Jiangsu Province, 213003, P.R. China; ^3^ State Key Laboratory for Medical Genomics, Shanghai Institute of Hematology, Collaborative Innovation Center of Hematology, National Research Center for translational Medicine, Ruijin Hospital, Shanghai Jiao Tong University School of Medicine, Shanghai 200025, China; ^4^ Institute of Oncology, Shanghai 9th People's Hospital, Shanghai Jiao Tong University School of Medicine, 639 Zhizaoju Rd, Shanghai 200011, China; ^5^ State Key Laboratory of Coordination Chemistry, Jiangsu Key Laboratory of Advanced Organic Materials, School of Chemistry and Chemical Engineering, Nanjing University, 163 Xianlin Avenue, Nanjing, Jiangsu 210023, China.

These authors contributed equally: Huizhuang Shan and Yang Cao.

* Correspondence to: Yingli Wu (E-mail:wuyingli@shsmu.edu.cn) or Zhujun Yao (E-mail: yaoz@nju.edu.cn).

**This file includes:**

Materials and Methods

Figures S1 to S11

Tables S1 to S2

References for supplementary materials

**Materials and Methods**

Cell culture and reagents

The human DLBCL cell lines OCI-Ly1, OCI-Ly3, OCI-Ly8, OCI-Ly10, DOHH2, and U2932 were kindly gifts from Dr. Rufang Xiang (Shanghai Jiao Tong University School of Medicine, Shanghai, China). SU-DHL-2, SU-DHL-4, and HEK293T cells were purchased from the American Type Culture Collection (ATCC, Manassas, VA, USA). OCI-Ly1, OCI-Ly3, OCI-Ly8, and OCI-Ly10 cells were cultured in Iscove’s Modified Dulbecco’s Medium (IMDM; Gibco, Waltham, MA, USA). The remaining four DLBCL cell lines were cultured in RPMI-1640 medium (BasalMedia, Shanghai, China). HEK293T cells were cultured in Dulbecco’s modified Eagle’s medium (DMEM; HyClone, Logan, UT, USA), which contains 10% fetal bovine serum (FBS; Gibco) and 1% penicillin-streptomycin (Gibco). All cells were kept in a 37 °C incubator with 5% CO_2_.

The synthesis of YL064 and biotin-labeled YL064 were described previously^1^. Both YL064 and sinomenine stock solution (20 mM) were maintained in dimethyl-sulfeoxide (DMSO; Sigma, St. Louis, MO, USA) and stored at -20 °C. MG132, MLN4924, and Stattic were purchased from CSNpharm (Chicago, IL, USA). Chloroquine (CQ) and cycloheximide (CHX) were purchased from Selleckchem (Houston, TX, USA). The BCL-2 inhibitor ABT-199 (venetoclax) was obtained from Chemshuttle (Jiangsu, China).

Quantitative real-time PCR (qRT-PCR)

Total RNA was first isolated from DLBCL cells employing TRIzol reagent (Invitrogen). Then the reverse transcription (RT) reaction was performed with an SYBR Premix Ex Taq II kit (TaKaRa, Shiga, Japan). The qRT-PCR was conducted using SYBR-Green qPCR Master Mix (Applied Biosystems, Foster City, CA, USA) in ABI 7900 Real-time PCR System (Applied Biosystems). All relevant primers sequences used in this study are stated as follows: MYC forward 5′-CCTGGTGCTCCATGAGGAGAC-3′ and MYC reverse 5′-CAGACTCTGACCTTTTGCCAGG-3′; MAX forward 5′-TGCACTGGAACGAAAACGTAG-3′ and MAX reverse 5′-GTCGTCAATATCTTGCTGGTGT-3′; β-actin forward 5′-CATCCTCACCCTGAAGTACCC-3′ and β-actin reverse 5′-AGCCTGGATAGCAACGTACATG-3′.

Cellular thermal shift assay (CETSA)

Cell lysates of OCI-Ly3 and SU-DHL-2 cells were obtained and incubated with YL064 (100 μM) or DMSO, respectively, and heated at different temperatures for 3 minutes. The mixtures were centrifuged, and the supernatants were subjected to SDS-PAGE and western blotting analysis. The dose effect of YL064 on the thermal stability of c-Myc was evaluated as follows. The cell lysates were obtained and aliquoted into 7 portions and incubated with various concentrations of YL064 for 30 min. Subsequently, the lysate solutions were heated at 45.4 °C for 3 min (Veriti thermal cycler, Applied Biosystems). The mixtures were centrifuged, and the supernatants were subjected to SDS-PAGE and western blotting analysis.

Plasmids, overexpression and transfection

The pcDNA3-Flag-c-Myc plasmid was obtained from Prof. Jianxiu Yu (Shanghai Jiao Tong University School of Medicine, Shanghai, China). The original wild-type HA-tagged c-Myc and its domain constructs^2^ were kindly provided by Prof. Wuhan Xiao (Institute of Hydrobiology, Chinese Academy of Sciences, Wuhan, China). Overexpression of c-Myc was performed using the pMSCV retroviral plasmid. Retroviral particles were produced in HEK293T cells by co-transfection of construct and packaging plasmid (pGag-Pol and pVSV-g), using the lipofectamine 3000 (Invitrogen), according to the manufacturer’s instructions. After 48 h, OCI-Ly3 and SU-DHL-2 cells were infected with viral supernatants and culture medium containing 8 μg/ml polybrene (Sigma).

Cell viability assay

The cell viability assay was performed using a Cell Counting Kit-8 (CCK-8) commercial kit (Dojindo, Kumamoto, Japan). DLBCL cells were plated in 96-well plates (2×10^3^ cells/well) and treated with different drugs for indicated doses. After incubation for indicated time-points, the incubation continued for another 2-4 h after the addition of 10 μL CCK-8 reagent. The absorbance at 450 nm determined using a microplate reader (BioTek Instruments, Winooski, VT, USA).

Flow cytometry analysis

The cell cycle analysis was evaluated using Propidium Iodide (PI; BD Biosciences, Franklin Lakes, NJ, USA) staining. Cell apoptosis was detected by AnnexinV-APC/PI assay apoptosis detection kit (BD) according to the manufacturer’s protocol. All the stained cells above were analyzed with flow cytometry (BD).

Preparation of recombinant wild-type c-Myc protein

Human full-length c-Myc were cloned into a pET-30c vector containing a 6×His tag at the C-terminal region. The plasmid was transformed into *Escherichia coli* BL21 (DE3) cells. c-Myc protein was then induced by IPTG and purified.

Immunofluorescence and TUNEL assay

OCI-Ly3 cells treated with biotin-YL064 or biotin were harvested and fixed for immunofluorescence staining. After permeabilization with 0.1% (v/v) Triton X-100 and blocking with 2% (w/v) BSA, the cells were incubated with an admixture of c-Myc antibody (1:50 dilution; Abcam) and streptavidin-FITC (1:100 dilution; eBioscience, San Diego, CA, USA) overnight at 4 °C. Then, the cells were stained with a TRITC-conjugated secondary antibody (1:200 dilution; Invitrogen) at room temperature for 1 h. Cellular DNA was counterstained with 4,6-diamidino-2-phenylindole (DAPI; Molecular Probes, Eugene, OR, USA). The fluorescence signals were obtained under a laser scanning confocal microscope (Nikon, Nagoya, Japan).

For TUNEL assay, DLBCL cells were exposed to DMSO or YL064 for 24 h, harvested, fixed, and permeabilized as mentioned above. Next, TUNEL detection buffer (Beyotime, Shanghai, China) was added and incubated at 37 °C for 1 h. Cellular DNA was counterstained with DAPI at room temperature for 5 min. The cells were imaged under a fluorescence microscope.

RNA-seq and data analysis

The total RNA samples from OCI-Ly3 and SU-DHL-2 cells were prepared with Trizol reagent (Invitrogen, USA) after treatment with YL064 or DMSO for 6 h. RNase-free DNase was employed to remove the contaminating DNA before RNA-seq. RNAs were sequenced by Shanghai Majorbio Biotechnology Co., Ltd., using Illumina HiSeq 4000 (Illumina, USA). The normalized read count data results were analyzed on the free online platform of Majorbio Cloud Platform (www.majorbio.com). Differential expression analysis (2-fold change, p-value < 0.001) was used to identify genes that significantly downregulated by YL064. Gene Set Enrichment Analysis (GSEA) was used to investigate specific gene sets associated with the differentially expressed genes between DMSO and drug-treated criteria. Sequencing and expression data have been deposited the Sequence Read Archive (SRA) with the accession number SRP250903.

Ubiquitination assay and western blotting

For ubiquitination assay, HEK293T cells were transfected with Flag-tagged c-Myc plasmid for 48 h. In order to assess the ubiquitination of c-Myc protein, the cells were treated with YL064 (20 μM for 6 h) in the presence or absence of MG132 (10 μM, added 4 h before the collection of cells). The cells were then lysed in lysis buffer containing protease inhibitors. After centrifugation at 12,000 rpm for 20 min at 4 °C, the supernatants were incubated with the anti-Flag M2 beads (Sigma) at 4 °C overnight. Next, the beads were washed with the lysis buffer more than three times. Finally, the bound proteins were eluted in SDS-PAGE sample buffer and analyzed by western blotting.

For western blotting, DLBCL cells were collected and lysed using SDS-PAGE sample buffer. Protein extracts were separated by SDS-PAGE gels (8-12%), followed by transferring to nitrocellulose membranes (Millipore, Danvers, MA, USA) and analyzed by western blotting. Antibodies against c-Myc (ab32072), c-Myc (pS62) (ab185656), c-Myc (pT58) (ab185655), vinculin (ab129002), streptavidin (ab7403) and β-Actin (ab8227) were purchased from Abcam (Cambridge, MA, USA). The Flag (#14793), HA(#3724), Ubiquitin (#43124), PARP (#9532), Cleaved caspase-3 (#9661), Caspase-3 (#9662), Caspase-9 (#9502), GSK-3ꞵ (#12456), STAT3 (#9139), p-STAT3 (Tyr705) (#9145), MCL-1 (#5453), BCL-2 (#4223), BIM (#2933), BAX (#2772) and BCL-XL (#2764) antibodies were obtained from Cell Signaling Technology (Dallas, TX, USA). All antibodies were diluted at 1:1000 in this experiment. Horseradish peroxidase (HRP)-conjugated secondary antibodies (Millipore) were used to detect the blots. The expression of the proteins was visualized by enhanced chemiluminescence (GE Healthcare, Piscataway, NJ, USA).

Immunoprecipitation

For mapping the direct binding region (s) of c-Myc protein to YL064, HEK293T cells were transfected with different mutant constructs and the full length of c-Myc. The cell lysates were incubated with biotin or biotin-YL064 at 4 °C overnight. The streptavidin beads (Smart-Lifesciences, Changzhou, China) then added for 4 h. Finally, the bound proteins were dissolved in SDS-PAGE loading buffer and analyzed by western blotting.

Assessment of YL064 and ABT-199 synergy

In combination studies, according to the sensitivity of the cells to each drug, DLBCL cells were treated with different combinations of YL064 and ABT-199 at indicated concentrations for 48 h. Combination index (CI) were calculated for the combinations of YL064 and ABT-199 using Compusyn (Combosyn Inc, Paramus, NJ) according to the Chou-Talalay algorithm.

Xenograft models

Five-week-old male NOD-*Prkdc*^em26^*Il2rg*^em26^/Gpt (NCG) or BALB/c nu/nu mice were obtained from GemPharmatech Co., Ltd and housed under standard conditions. For the OCI-Ly3 xenograft model, 4 × 10^6^ tumor cells were mixed with Matrigel (Corning, Bedford, MA, USA) at a 1:1 ratio and injected subcutaneously into the right flanks of NCG mice. For the OCI-Ly8 xenograft model, 8 × 10^6^ tumor cells were injected subcutaneously into the right flanks of BALB/c nu/nu mice. When the tumors became palpable, the mice were randomly divided into 4 groups and treated with: (1) YL064, intraperitoneally, 30 mg/kg/day ; (2) ABT-199, orally, 50 mg/kg, 5 days on/2 days off; (3) both drugs at indicated doses; or (4) vehicles: 8% DMSO, 10% 1,2-Propanediol, 10% Cremophor ELP, and 72% normal saline for YL064 and 5% DMSO, 10% Cremophor EL, and 85% PBS for ABT-199. Tumor volumes and body weight were assessed every other day. At the end of the animal experiment, the tumors were isolated, photographed, and weighed. Tumor volume was calculated as formula V = a × b^2^/2, where a and b are the length and the width of tumor, respectively. Then, the samples were fixed in 4% paraformaldehyde and processed for paraffin sectioning. All animal experiments were conducted under the approval of the Experimental Animal Ethical Committee at Shanghai Jiao Tong University School of Medicine.

Statistical analysis

The data from at least three independent experiments were exhibited as mean ± SD or SEM. The data were analyzed using either Student’s *t*-test (two-group comparison) or one-way *ANOVA* (more than two groups). A *p*-value less than 0.05 was considered statistically significant (^*^*p* < 0.05; ^**^*p* < 0.01; ^***^ *p* < 0.001; ^****^ *p* < 0.0001). All graphs were generated using GraphPad Prism 8.0 software (GraphPad Software Inc., La Jolla, CA, USA).

**Figure. S1**


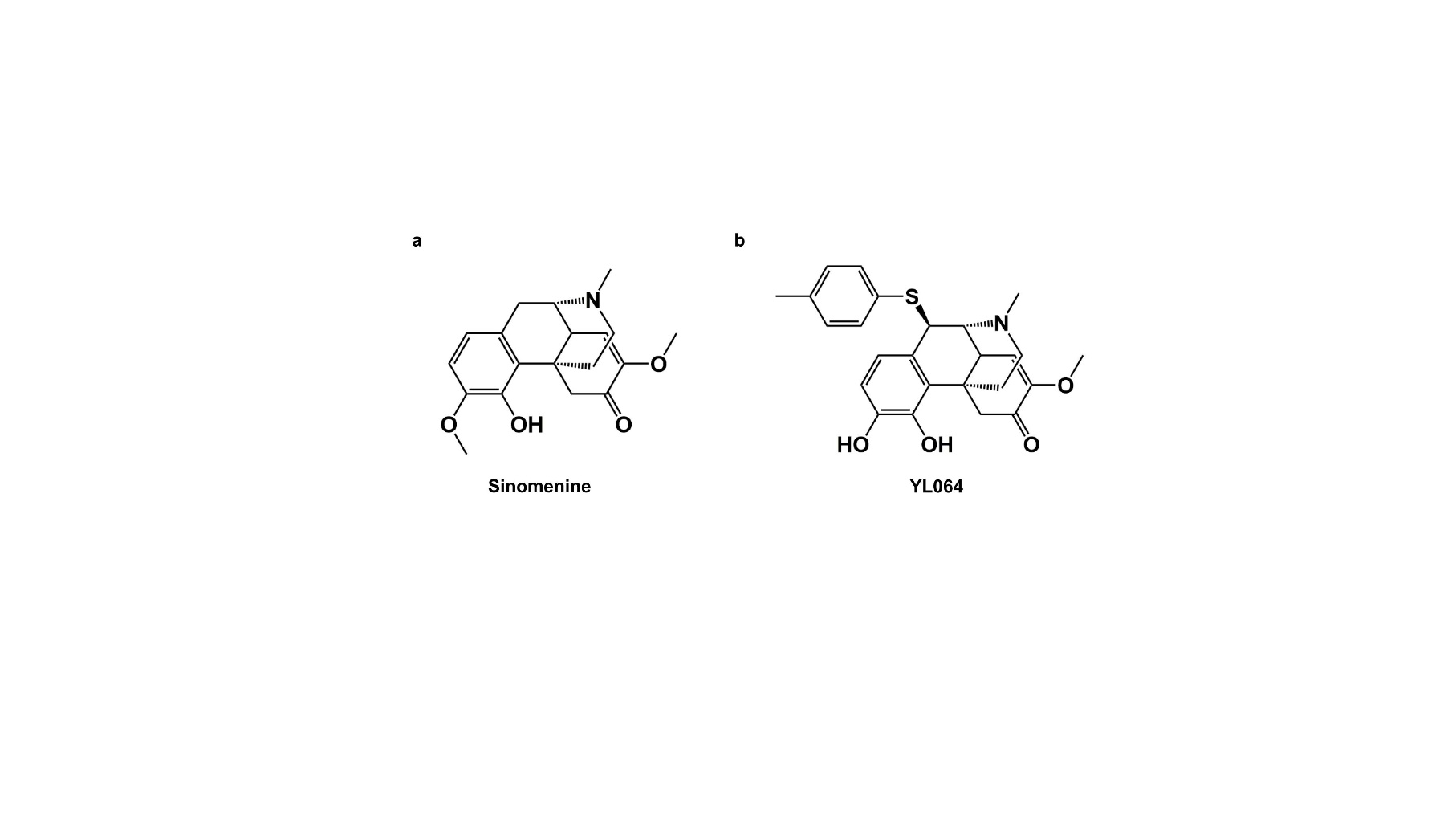


Chemical structure of sinomenine (a), and YL064 (b).

**Figure. S2**


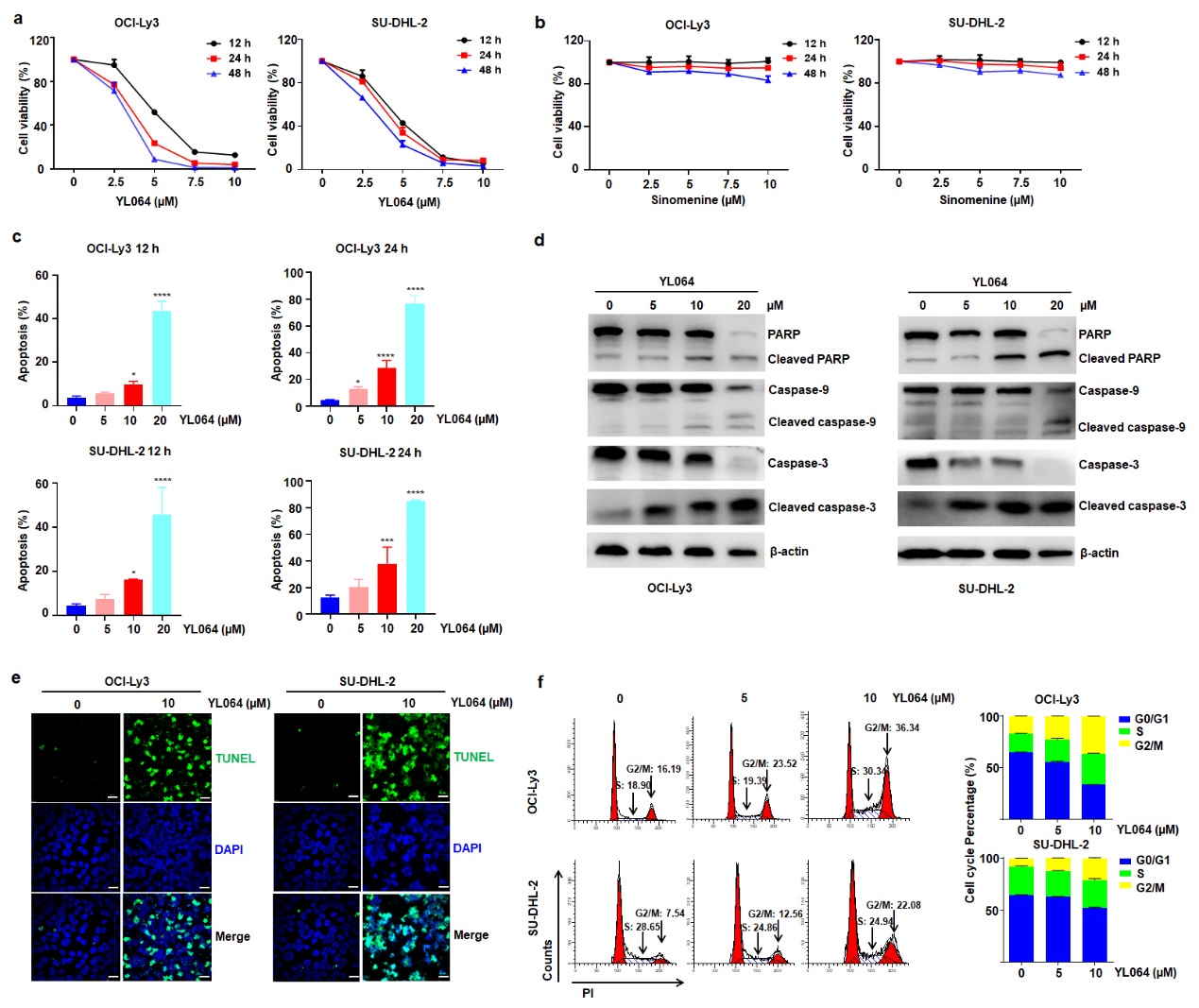


(a, b) OCI-Ly3 and SU-DHL-2 cells were treated with the indicated concentrations of YL064 (a) or sinomenine (b) for the indicated time. Cell viability was determined by CCK-8 assay. (c) OCI-Ly3 and SU-DHL-2 cells were treated with YL064 (0-20 μM) for 12 and 24 h. Cell apoptosis was analyzed by flow cytometry using Annexin V/PI staining. Columns represent the average percent of Annexin V positive cells from three independent experiments, which are shown as the mean ± SD. ^*^*p* < 0.05, ^***^ *p* < 0.001, and ^****^ *p* < 0.0001. (d) OCI-Ly3 and SU-DHL-2 cells were treated with YL064 (0-20 μM) for 24 h. Whole cell lysates were subjected to western blotting using indicated antibodies. (e) Apoptosis analyzed by TUNEL assay. Scale bars are 20 μm. (f) Cell cycle analysis of DLBCL cells treated with YL064 for 12 h. Columns (right panel) represent cell cycle distribution in DLBCL cells. Results are representative of three repetitions.

**Figure. S3**

**
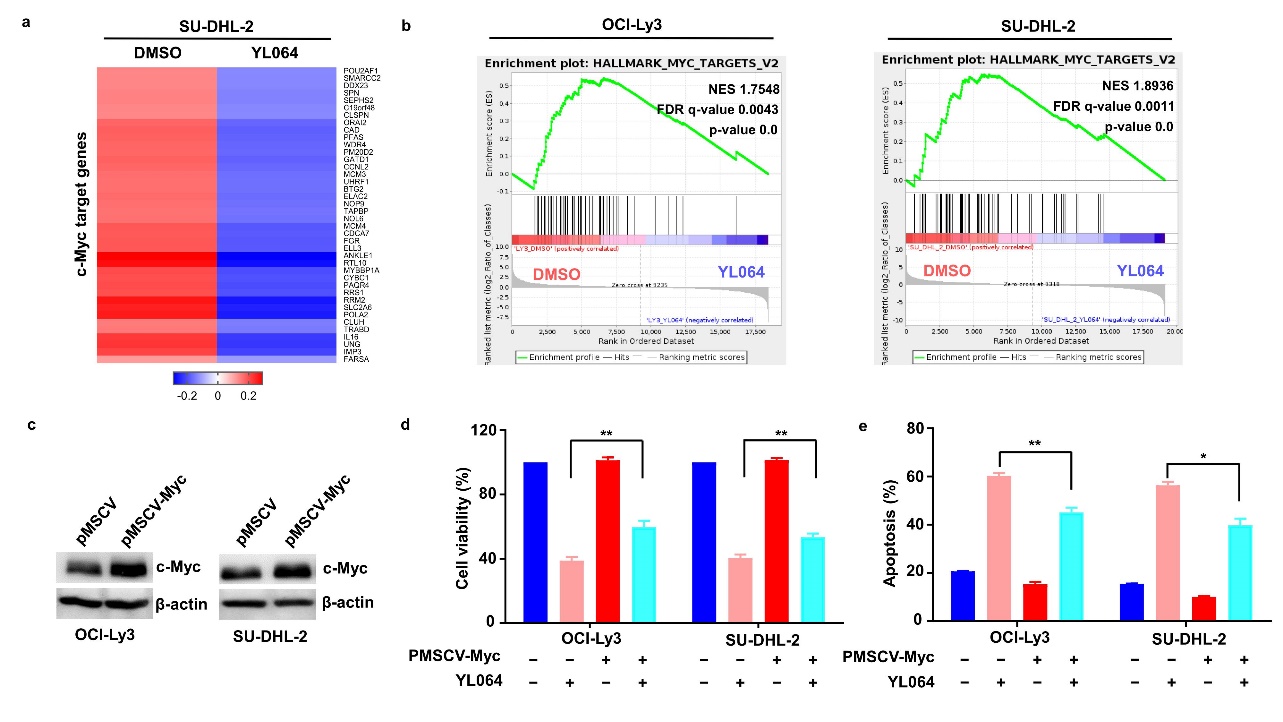
**

(a) Heatmap of the top 40 significantly downregulated genes for c-Myc targets in SU-DHL-2 cells treated with YL064 (10 μM) versus DMSO for 6 h. (b) GSEA showing MYC gene signatures in OCI-Ly3 and SU-DHL-2 cells after treatment with YL064 (versus DMSO).(c-e) OCI-Ly3 and SU-DHL-2 cells were transiently transfected with empty pMSCV vector or c-Myc overexpression vector. (c) The c-Myc overexpression cell lines were evaluated by western blotting analysis for indicated antibodies. (d) After 24 h of treatment of 5 μM YL064, cell viability was measured by CCK-8 assay. (e) After 24 h of treatment of 10 μM YL064, cell apoptosis was analyzed by flow cytometry using Annexin V/PI staining. All values represent the means ± S.D. of three independent experiments. ^*^*p* < 0.05, ^**^ *p* < 0.01, and ^***^ *p* < 0.001.

**Figure. S4**

**
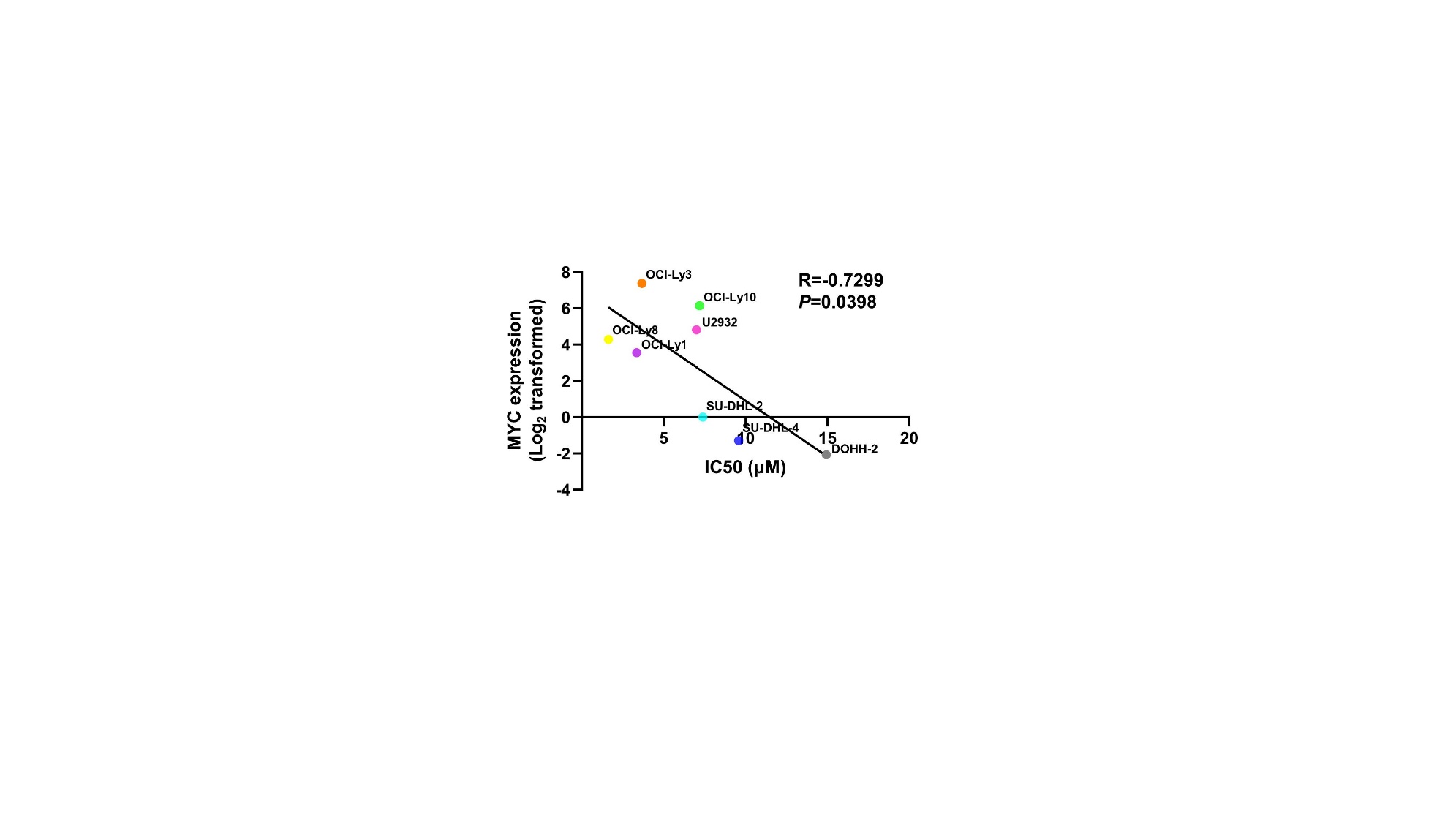
**

Correlation analysis between IC_50_ values of YL064 and relative mRNA level of MYC among eight DLBCL cell lines using GraphPad Prism software.

**Figure. S5**


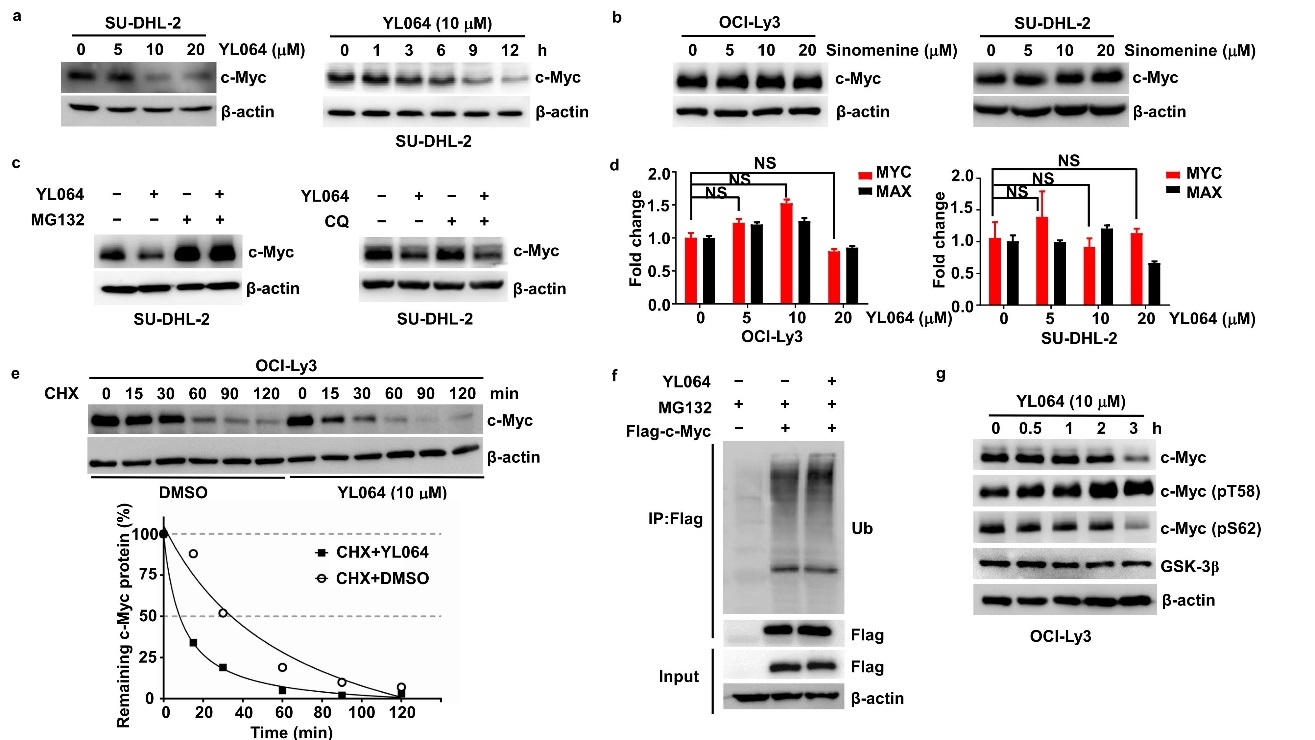


(a) SU-DHL-2 cells were treated with the indicated concentrations of YL064 for 12 h (left panel) or YL064 (10 μM) for the indicated time points (right panel). Protein expression was quantified by western blotting. (b) OCI-Ly3 and SU-DHL-2 cells were treated with the indicated concentrations of sinomenine for 12 h and analyzed by western blotting. (c) OCI-Ly3 and SU-DHL-2 cells were treated with YL064 (10 μM) in the presence or absence of MG132 (5 μM, left panel) or chloroquine (20 μM, right panel) for 6 h, the indicated proteins were examined by western blotting. (d) OCI-Ly3 and SU-DHL-2 cells were treated with increasing concentrations of YL064 for 12 h. The mRNA level of MYC and MAX were examined by qRT-PCR. Data represent mean ± SD and NS denotes no significant. (e) OCI-Ly3 cells were preincubated with either DMSO or YL064 (10 μM, 3 h), followed by CHX (10 μg/mL) treatment for up to 2 h. Cells were harvested at the indicated time points and analyzed for c-Myc expression using western blotting. (f) HEK293T cells were transfected with Flag-tagged c-Myc plasmid, treated with or without YL064 (20 μM) for 6 h before cells were harvested. Cellular extracts were prepared for immunoprecipitation assays with anti-Flag followed by western blotting with anti-Ub and anti-Flag. (g) Western blotting for c-Myc, phosphorylated c-Myc T58 and S62, and GSK-3ꞵ in YL064-treated OCI-Ly3 cells at the indicated time points.

**Figure. S6**

**
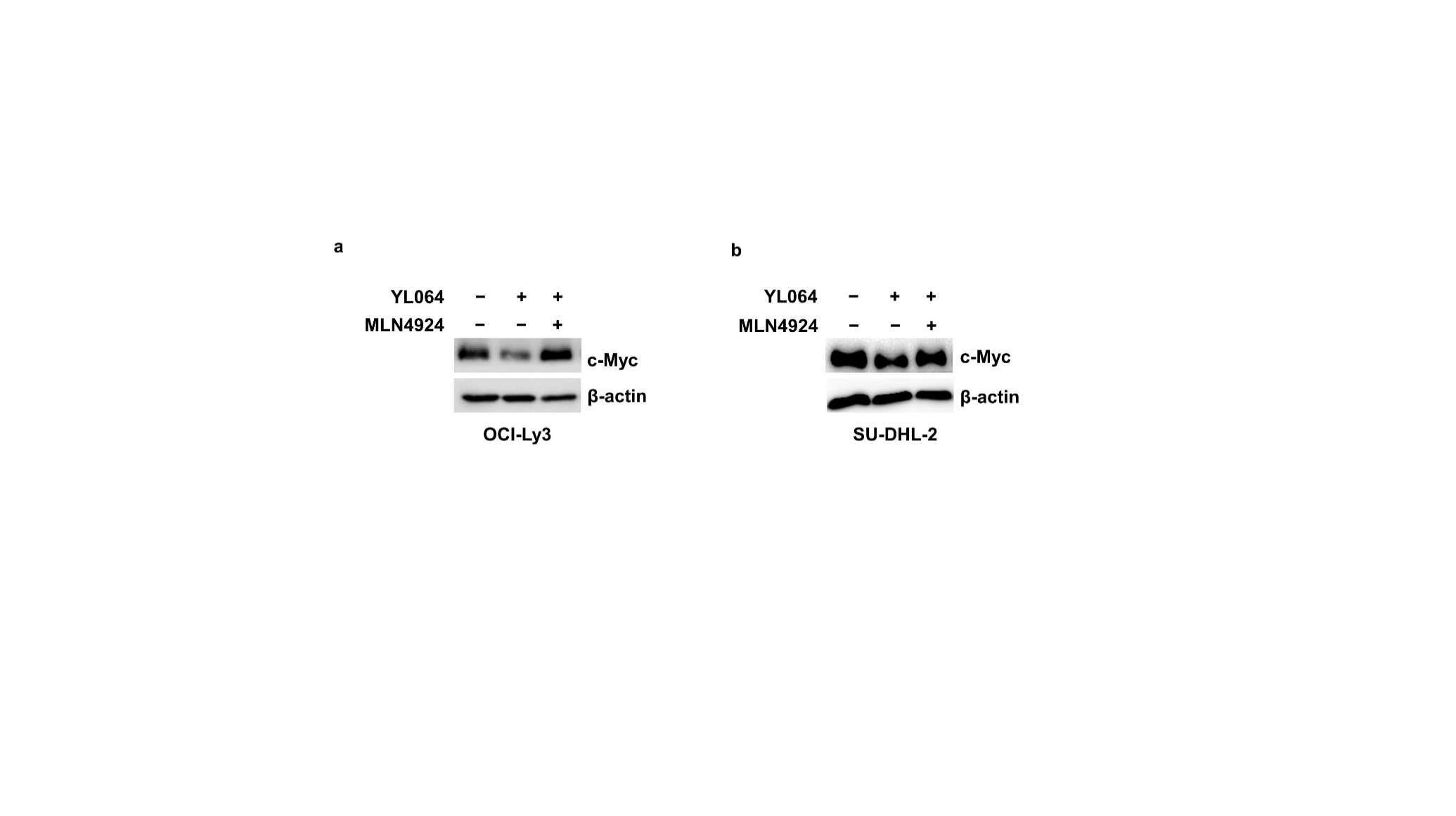
**

OCI-Ly3 and SU-DHL-2 cells were pretreated with 250 nM of MLN4924 for 2 h, followed by 6 h of treatment with 10 μM YL064. The whole cell lysates were subjected to western blotting against the specific antibodies as indicated.

**Figure. S7**

**
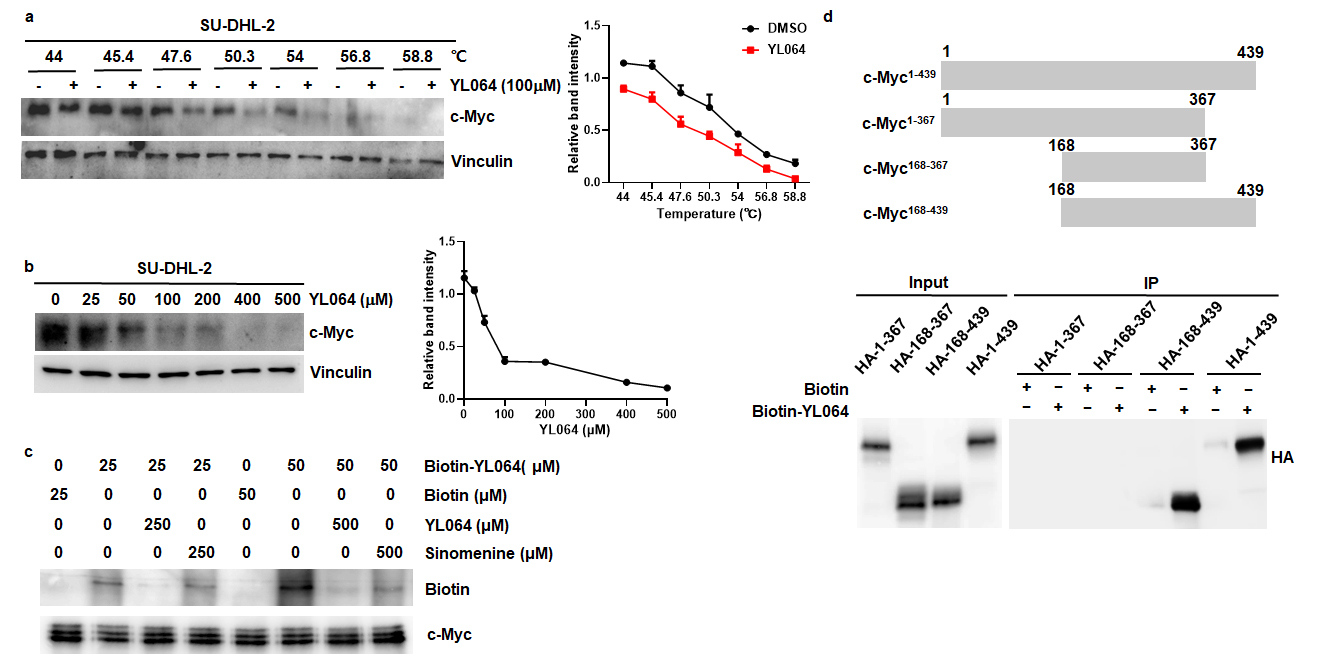
**

(a, b) The binding between YL064 and c-Myc protein in SU-DHL-2 cells was examined by the CETSA method at temperatures (upper panel) or doses (lower panel). The indicated proteins were evaluated by western blotting. The intensity of the c-Myc bands was quantified by Image J software. (c) The recombinant c-Myc protein was incubated with biotin-YL064 in the absence or presence of a ten-fold excess of unlabeled YL064 or sinomenine for 30 min, and the mixtures were subjected to western blotting for biotin or c-Myc. (d) Upper panel, a schematic representation of various c-Myc truncated mutants. Lower panel, binding effect between biotin-YL064 and the full-length or truncated c-Myc constructs (HA-tagged) was detected by immunoprecipitation assays followed by western blotting with anti-HA.

**Figure. S8**

**
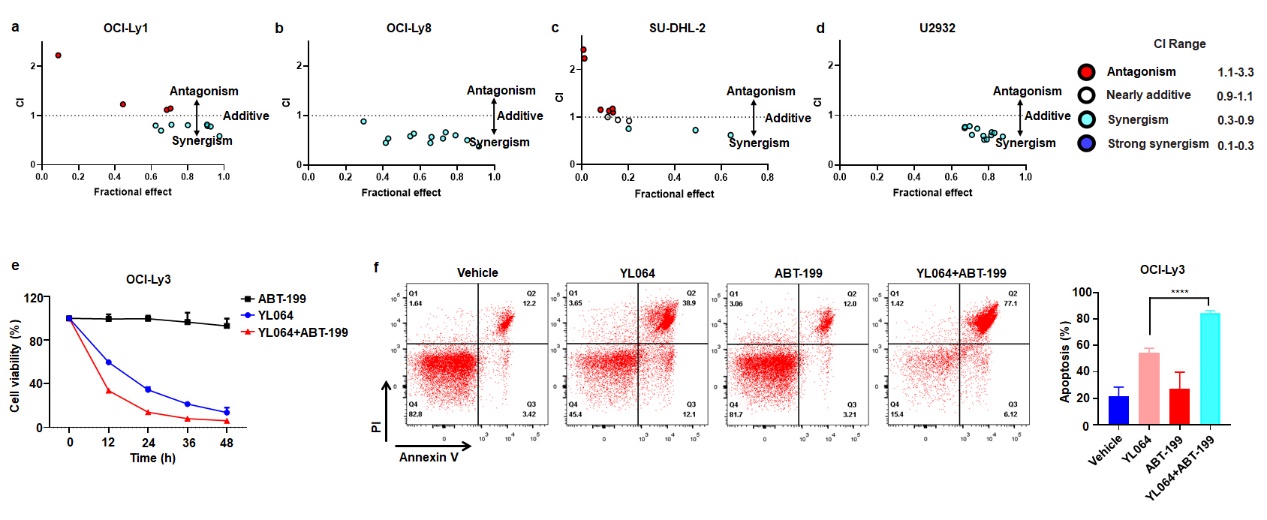
**

(a-d) DLBCL cell lines (OCI-Ly1, OCI-Ly8, SU-DHL-2, and U2932) were treated with the indicated concentration of YL064 and ABT-199, alone and in combination for 48 h. Combination index (CI) for each combination were calculated with the data obtained from the CCK-8 assays with the Calcusyn program (a CI of 1 indicates an additive effect, CI < 1 a synergistic effect and CI > 1antagonism). (e) OCI-Ly3 cells were treated with YL064 (5 μM) or ABT-199 (2 µM) and their combination for 12, 24, 36, and 48 h, respectively. Cell viability was determined by CCK-8 assay. (f) Representative data for flow cytometric analysis of Annexin V/PI staining in OCI-Ly3 cells after exposure to YL064 (10 μM), ABT-199 (2 µM) or YL064 plus ABT-199 for 24 h. Columns represent the average percent of Annexin V positive cells from three independent experiments, which are shown as the mean ± SD. ^****^ *p* < 0.0001. All results are representative of triplicate treatments.

**Figure. S9**


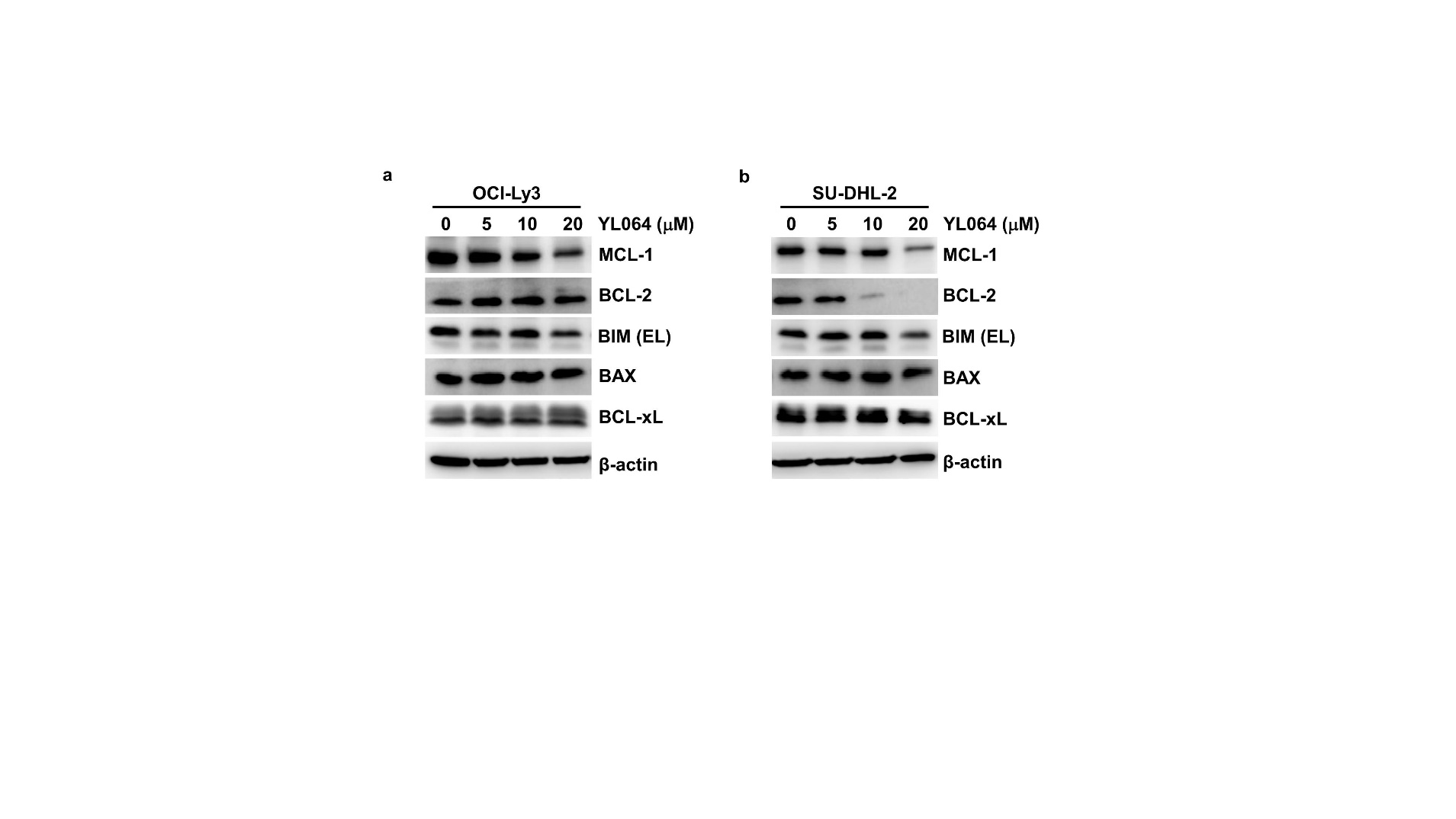


OCI-Ly3 (a) and SU-DHL-2 (b) cells were treated with the indicated concentrations of YL064 for 12 h and analyzed by western blotting against the BCL-2 family proteins as indicated.

**Figure. S10**

**
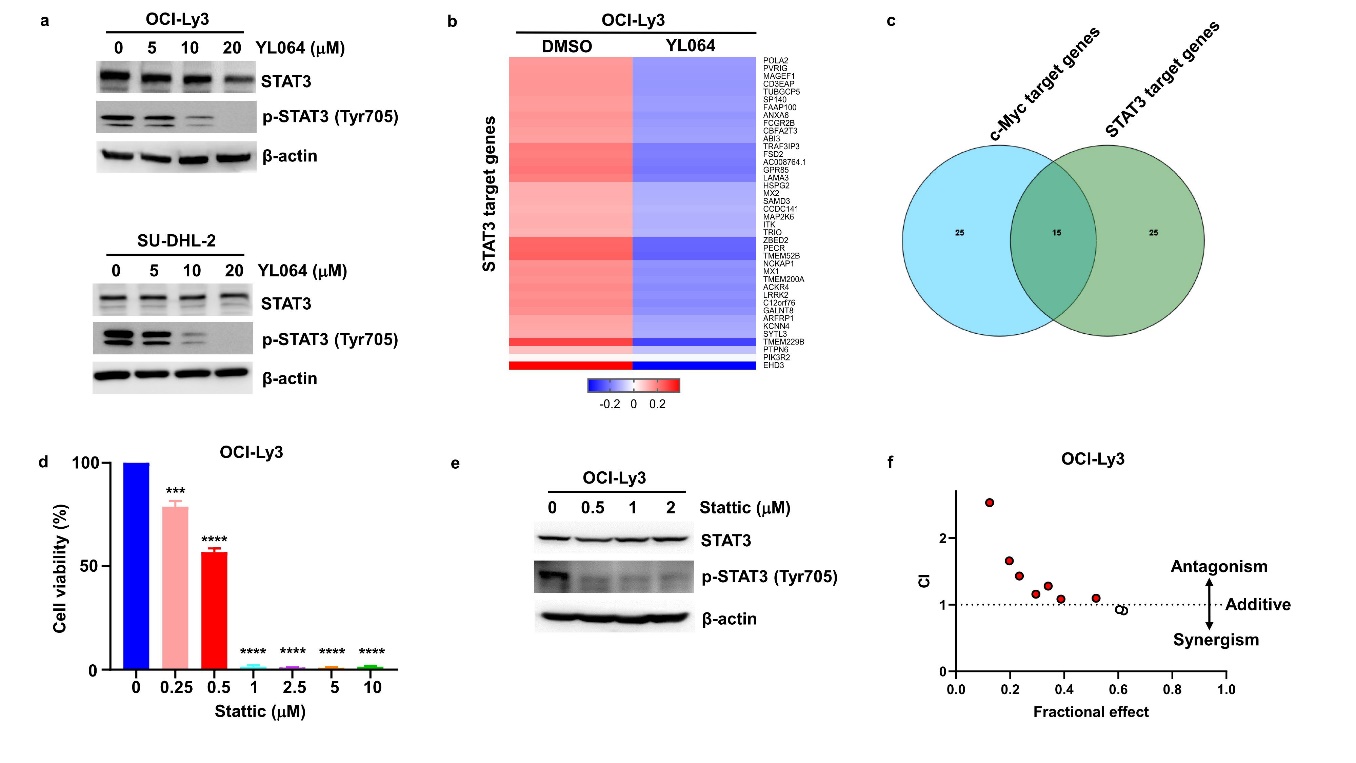
**

(a) OCI-Ly3 and SU-DHL-2 cells were treated with the indicated concentrations of YL064 for 12 h and analyzed by western blotting for indicated antibodies. (b) Heatmap of the top 40 significantly downregulated genes for STAT3 targets in OCI-Ly3 cells treated with YL064 (10 μM) versus DMSO for 6 h. (c) Venn diagram depicting the overlap between the top 40 significantly downregulated c-Myc and STAT3 target gene sets (YL064 versus DMSO) in OCI-Ly3 cells displayed in Fig. 1b and Fig. S10b. (d) OCI-Ly3 cells were treated with the indicated concentrations of Stattic for 48 h and the cell viability was tested by CCK-8 assay. ^***^ *p* < 0.001, ^****^ *p* < 0.0001. (e) OCI-Ly3 cells were treated with the indicated concentrations of Stattic for 48 h and detected by western blotting for indicated antibodies. (f) OCI-Ly3 cells were treated with the indicated concentration of Stattic and ABT-199, alone and in combination for 48 h. Combination index (CI) for each combination were calculated with the data obtained from the CCK-8 assays with the Calcusyn program.

**Figure. S11**

**
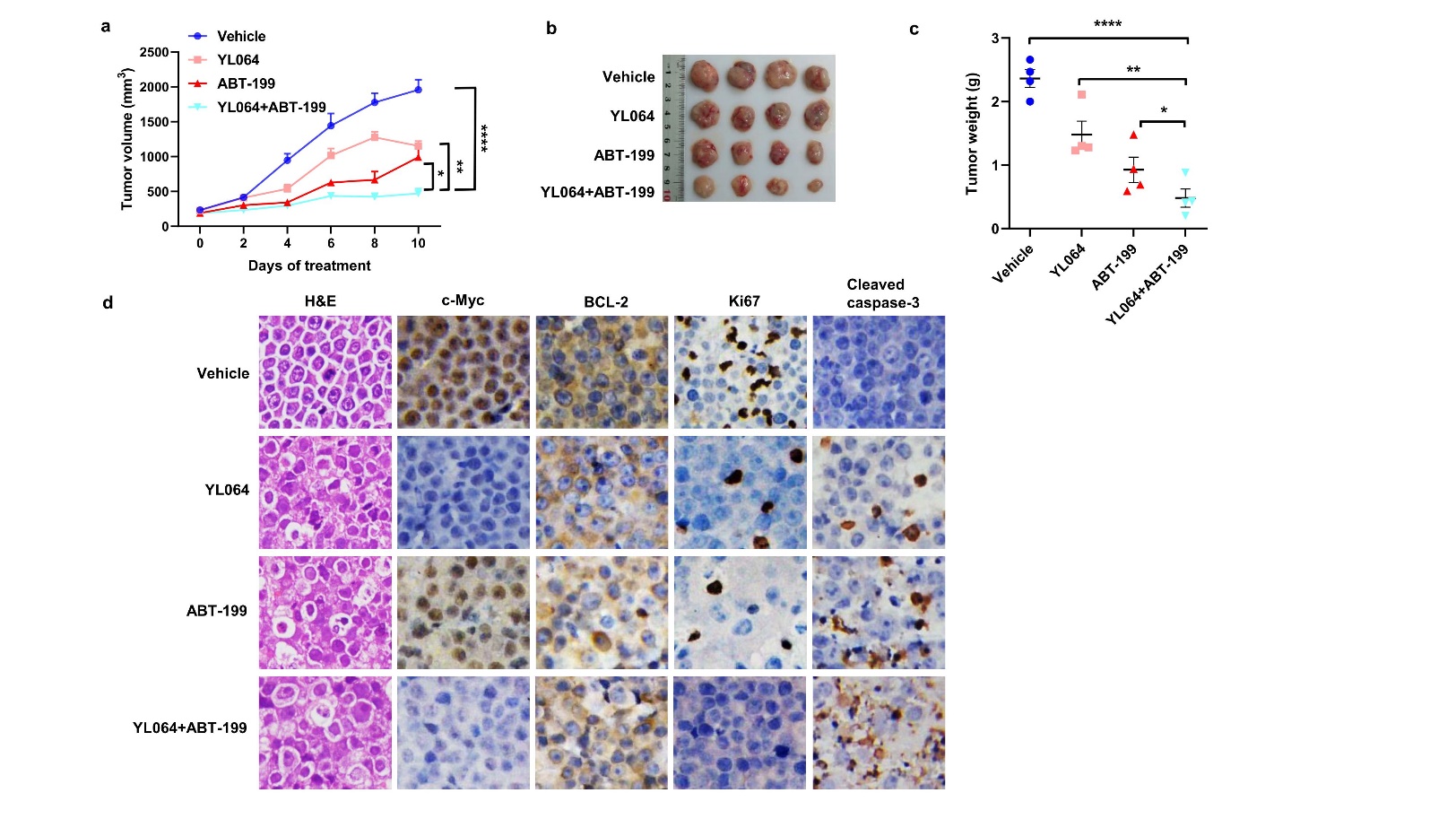
**

OCI-Ly8 cells were xenografted in mice as described in Materials and methods. Mice were treated with vehicle, 30 mg/kg YL064, 50 mg/kg ABT-199, or both drugs. (a) Tumor volume was measured every 2 days. (b) Images of mice (n = 4) from each group are presented to show the sizes of the tumors. (c) Tumors were excised from the animals and weighed. (d) Representative H&E staining and immunohistochemical staining of c-Myc, BCL-2, Ki67, and cleaved caspase-3 in tumor sections (original magnification × 400). Data are shown as means ± SEM. ^*^*p* < 0.05, ^**^ *p* < 0.01, and ^****^ *p* < 0.0001.

**Table S1.** **CI values of YL064 and ABT-199 in DLBCL cells**

| Cell line | Conc. (μM) YL064 | Conc. (nM) ABT-199 | CI |
| --- | --- | --- | --- |
| OCI-Ly1 | 2 | 2.5 | 0.69667 |
|  | 2 | 5 | 0.79965 |
|  | 2 | 10 | 0.81666 |
|  | 2.5 | 2.5 | 2.22579 |
|  | 2.5 | 5 | 1.23085 |
|  | 2.5 | 10 | 0.80797 |
|  | 3.5 | 2.5 | 1.11823 |
|  | 3.5 | 5 | 1.14603 |
|  | 3.5 | 10 | 0.78998 |
|  | 4 | 2.5 | 0.81908 |
|  | 4 | 5 | 0.78115 |
|  | 4 | 10 | 0.58349 |
| OCI-Ly3 | 2.5 | 1000 | 2.67028 |
|  | 2.5 | 1500 | 0.65219 |
|  | 2.5 | 2000 | 0.41634 |
|  | 3 | 1000 | 0.31464 |
|  | 3 | 1500 | 0.25112 |
|  | 3 | 2000 | 0.24495 |
|  | 3.5 | 1000 | 0.16956 |
|  | 3.5 | 1500 | 0.28677 |
|  | 3.5 | 2000 | 0.33045 |
|  | 4 | 1000 | 0.78398 |
|  | 4 | 1500 | 1.03868 |
|  | 4 | 2000 | 0.80276 |
| OCI-Ly8 | 0.5 | 12.5 | 0.45272 |
|  | 0.5 | 25 | 0.58708 |
|  | 0.5 | 50 | 0.66526 |
|  | 1 | 12.5 | 0.54113 |
|  | 1 | 25 | 0.63826 |
|  | 1 | 50 | 0.60552 |
|  | 1.5 | 12.5 | 0.88434 |
|  | 1.5 | 25 | 0.57085 |
|  | 1.5 | 50 | 0.50743 |
|  | 2 | 12.5 | 0.45083 |
|  | 2 | 25 | 0.53962 |
|  | 2 | 50 | 0.38001 |
| U2932 | 3.5 | 1000 | 0.51437 |
|  | 3.5 | 1500 | 0.60923 |
|  | 3.5 | 2000 | 0.51802 |
|  | 4 | 1000 | 0.58964 |
|  | 4 | 1500 | 0.7471 |
|  | 4 | 2000 | 0.77112 |
|  | 4.5 | 1000 | 0.60307 |
|  | 4.5 | 1500 | 0.78523 |
|  | 4.5 | 2000 | 0.73681 |
|  | 5 | 1000 | 0.66389 |
|  | 5 | 1500 | 0.6484 |
|  | 5 | 2000 | 0.57581 |
| SU-DHL-2 | 3.5 | 1000 | 2.42116 |
|  | 3.5 | 1500 | 2.23755 |
|  | 3.5 | 2000 | 0.75551 |
|  | 4 | 1000 | 1.00327 |
|  | 4 | 1500 | 1.15286 |
|  | 4 | 2000 | 0.93976 |
|  | 4.5 | 1000 | 0.92114 |
|  | 4.5 | 1500 | 1.12957 |
|  | 4.5 | 2000 | 1.10241 |
|  | 5 | 1000 | 1.17405 |
|  | 5 | 1500 | 0.72469 |
|  | 5 | 2000 | 0.61933 |

**Table S2. T****he significantly downregulated genes uniquely regulated by c-Myc (YL064 versus DMSO) in OCI-Ly3 cells related to Fig. S10c**

| Gene_ID | Gene name | P-value | FDR | Log_2_FC | Significant | Regulate |
| --- | --- | --- | --- | --- | --- | --- |
| ENSG00000136929 | HEMGN | 7.29E-24 | 1.75E-22 | -2.07618 | yes | down |
| ENSG00000037897 | METTL1 | 2.72E-22 | 5.98E-21 | -1.72962 | yes | down |
| ENSG00000173085 | COQ2 | 7.48E-20 | 1.4E-18 | -1.12091 | yes | down |
| ENSG00000185105 | MYADML2 | 3.44E-19 | 6.18E-18 | -1.00796 | yes | down |
| ENSG00000232838 | PET117 | 9.58E-15 | 1.29E-13 | -1.82562 | yes | down |
| ENSG00000171757 | LRRC34 | 3.8E-12 | 4.13E-11 | -1.21604 | yes | down |
| ENSG00000172346 | CSDC2 | 3.7E-10 | 3.41E-09 | -1.25175 | yes | down |
| ENSG00000267216 | AC020915.1 | 1.07E-09 | 9.36E-09 | -2.30062 | yes | down |
| ENSG00000140092 | FBLN5 | 5.58E-09 | 4.54E-08 | -1.46354 | yes | down |
| ENSG00000132746 | ALDH3B2 | 8.72E-09 | 6.94E-08 | -1.35751 | yes | down |
| ENSG00000106003 | LFNG | 2.33E-08 | 1.77E-07 | -2.11682 | yes | down |
| ENSG00000128536 | CDHR3 | 3.62E-08 | 2.7E-07 | -1.12474 | yes | down |
| ENSG00000258643 | BCL2L2-PABPN1 | 3.83E-08 | 2.84E-07 | -5.81222 | yes | down |
| ENSG00000213533 | STIMATE | 1.45E-07 | 1.01E-06 | -2.82162 | yes | down |
| ENSG00000176273 | SLC35G1 | 1.96E-07 | 1.34E-06 | -1.23967 | yes | down |
| ENSG00000266074 | BAHCC1 | 2.22E-07 | 1.51E-06 | -2.56748 | yes | down |
| ENSG00000248919 | ATP5MF-PTCD1 | 2.55E-07 | 1.72E-06 | -1.00326 | yes | down |
| ENSG00000111913 | RIPOR2 | 2.83E-07 | 1.89E-06 | -2.04476 | yes | down |
| ENSG00000167774 | AC010323.1 | 8.45E-07 | 5.33E-06 | -1.07107 | yes | down |
| ENSG00000279152 | AC129492.8 | 1.17E-06 | 7.24E-06 | -2.21069 | yes | down |
| ENSG00000162746 | FCRLB | 1.36E-06 | 8.38E-06 | -1.04 | yes | down |
| ENSG00000131187 | F12 | 5.04E-06 | 2.88E-05 | -3.13276 | yes | down |
| ENSG00000188613 | NANOS1 | 5.57E-06 | 3.17E-05 | -1.02988 | yes | down |
| ENSG00000205923 | CEMP1 | 7.29E-06 | 4.09E-05 | -2.2499 | yes | down |
| ENSG00000257921 | AC025165.3 | 7.71E-06 | 4.31E-05 | -3.78161 | yes | down |

**References**

1. Wang, Y. *et al.* YL064 directly inhibits STAT3 activity to induce apoptosis of multiple myeloma cells. *Cell Death Discov* **4**, 44 (2018).

2. Chen, Y. *et al.* ELL targets c-Myc for proteasomal degradation and suppresses tumour growth. *Nat Commun* **7**, 11057 (2016).
